# Role of Lipid-Based and Polymer-Based Non-Viral Vectors in Nucleic Acid Delivery for Next-Generation Gene Therapy

**DOI:** 10.3390/molecules25122866

**Published:** 2020-06-22

**Authors:** Aniket Wahane, Akaash Waghmode, Alexander Kapphahn, Karishma Dhuri, Anisha Gupta, Raman Bahal

**Affiliations:** 1 Department of Pharmaceutical Sciences, University of Connecticut, Storrs, CT 06269, USA; aniket.wahane@uconn.edu (A.W.); karishma.dhuri@uconn.edu (K.D.); 2 Department of Chemistry, Wesleyan University, Middletown, CT 06459, USA; awaghmode@wesleyan.edu (A.W.); akapphahn@wesleyan.edu (A.K.)

**Keywords:** gene therapy, nanoparticles, lipid nanoparticles, nucleic acids, mRNA, DNA

## Abstract

The field of gene therapy has experienced an insurgence of attention for its widespread ability to regulate gene expression by targeting genomic DNA, messenger RNA, microRNA, and short-interfering RNA for treating malignant and non-malignant disorders. Numerous nucleic acid analogs have been developed to target coding or non-coding sequences of the human genome for gene regulation. However, broader clinical applications of nucleic acid analogs have been limited due to their poor cell or organ-specific delivery. To resolve these issues, non-viral vectors based on nanoparticles, liposomes, and polyplexes have been developed to date. This review is centered on non-viral vectors mainly comprising of cationic lipids and polymers for nucleic acid-based delivery for numerous gene therapy-based applications.

## 1. Introduction

**Figure 1:**
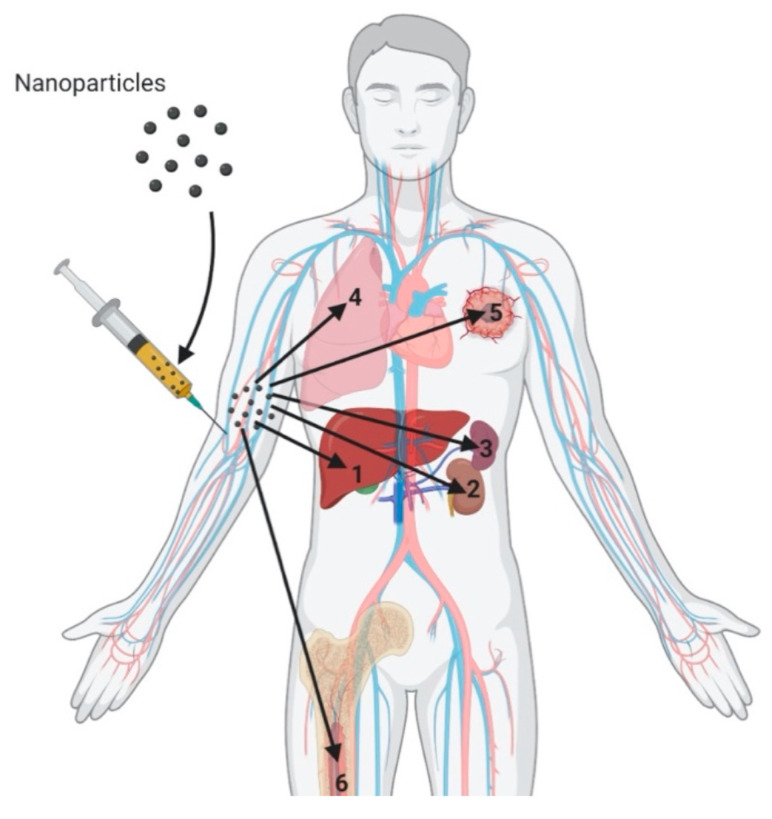
Physiological barriers for nanocarrier delivery following systemic administration. 1. Nanocarriers predominately accumulate in liver due to their size and velocity of blood flow. Nanoparticle accumulation in liver and rapid clearance is mainly governed by liver Kupffer cells that form the mononuclear phagocytic system (MPS) [8]. 2. Renal clearance is due to their small particle size (less than 8 nm). Larger nanocarriers are redirected for hepatic clearance [8]. 3. Spleen is the other organ that constitutes MPS and causes accumulation as well as nanocarrier clearance [8]. 4. The presence of a thick mucosal layer in the lungs acts as a barrier for targeted pulmonary delivery. Macrophages in the lungs also contribute to the accumulation and clearance of nanoparticles. 5. Nanocarriers of small particle sizes (<200 nm) target the tumors due to enhanced permeability and retention (EPR) effect caused by leaky vasculature [9]. 6. Macrophages that reside in bone marrow are also a part of the MPS system, leading to nanocarrier accumulation and clearance [8].

Gene therapy has gained considerable attention in the last few years for treating myriads of devastating diseases. Gene therapy relies on the introduction of nucleic acids or their synthetic analogs (also called nucleic acid analogs) or genome editing proteins (especially nucleases), in the cells to regulate gene expression with minimal off-target toxicity. Thus, gene therapy resolves the problem at its source. Though many advances have been made in the field of gene therapy, the delivery of nucleic acid analogs (NAA), as well as genome editing proteins to the target site, continues to be an unresolved issue for the broad utility of gene therapy-based applications. The delivery must be assisted with synthetic biocompatible nanocarriers to overcome the biological barriers and to deliver the cargo at the site of action [1]. A summary of the physiological barriers that nanocarriers must overcome following systemic circulation is illustrated in Figure 1. Synthetic polymer-based nanocarriers have shown promise in this regard. They are easy to synthesize and scale up for clinical applications. However, delivery systems possess inherent challenges, including low payload [2], endosomal entrapment [3], enzymatic degradiation [4], and short bioavailability time [5]. Hence, to overcome these limitations, cationic carrier systems have been tested. In general, the cationic carrier enables the optimal entrapment and condensation of small molecules as well as nucleic acids, and it forms stable complexes without affecting their integrity [6]. Presently, two widely used cationic carrier systems, cationic lipids and cationic polymers, are being examined [7]. This review exclusively summarizes a new generation of cationic lipids and cationic polymers and their conjugates for the delivery of nucleic acids, their analogs, as well as genome editing proteins for diverse therapeutic applications.

## 2. Lipid-Based Vectors

Liposome formulations have been used widely for the delivery of small molecules and macromolecule-based therapeutics [10]. Liposomes are spherical delivery systems with hydrophilic polar head groups and hydrophobic tails. Liposomes can efficiently encapsulate water-soluble agents in their hydrophilic core and water-insoluble agents in their lipid membrane [10]. N-[1-(2,3-dioleyloxy)propyl]-N,N,N-trimethylammonium chloride (DOTMA) was the first cationic lipid used to form liposomes. It showed approximately 100% entrapment of plasmid DNA with 5 to 100-fold transfection efficiency as compared to calcium phosphate and diethylaminoethyl-dextran in a variety of cell culture assays [11]. DOTMA-based liposomes form complexes with nucleic acids due to electrostatic interaction and are also known as lipoplexes. Several efforts have been made to understand the entrapment and release of nucleic acids from the lipid bilayers [12] alongside the optimization of lipid chemistry to form stable optimum sized liposomes [13]. However, due to their cationic nature, liposomes bind non-specifically to serum proteins and cause toxicity [14]. This has led to the use of helper lipids such as 1,2-dioleoyl-sn-glycero-3-phosphoethanolamine (DOPE) [15], cholesterol (CHO) [16,17], phosphatidylcholines (PCs) [18,19], and polyethylene glycol (PEG)ylating lipids [20,21] which tend to reduce the surface charge of cationic lipids, provide stability to lipid nanoparticles (LNPs), and reduce reticuloendothelial system (RES) uptake to increase their blood circulation time [22].

Liposomal vectors based on DOTMA were the first generation of lipid-based vectors utilized to deliver plasmid DNA in vitro. However, DOTMA-based formulations also caused cellular toxicity along with the activation of the immune system because of their cationic charge [23]. Hence, DOTMA-based formulations cannot be applied to various in vivo studies [24]. This further led to the exploration of helper lipids and lipids other than phospholipids to achieve smaller particle size and acceptable tolerability. Lipid nanoparticles (LNPs) are different than liposomes as they incorporate ionizable lipids to entrap nucleic acids and do not have an aqueous core. Wheeler et al. first explored LNPs using stabilized plasmid lipid particles (SPLPs) using a detergent dialysis method. Dioleoyl-dimethyl-ammonium chloride (DODAC) as a cationic lipid with 1,2-dioleoyl-sn-glycero-3-phosphoethanolamine (DOPE) as a helper lipid and polyethylene glycol (PEG) were used to encapsulate plasmid cytomegalovirus chloramphenicol transferase (pCMVCAT). This generated small uniform particles (approximately 70 nm) with optimal encapsulation efficiency (70%) [25]. A thorough exploration of different PEG lipids yielded SPLPs with longer blood circulation time. Similarly, SPLPs encapsulating pLuc (plasmid luciferase) avoided organs exhibiting first-pass effect (liver and spleen) that resulted in 100 to 1000-fold gene expression levels in distal tumor tissues following intravenous (IV) administration in a murine tumor model [26]. Furthermore, in a comparative study between liposomal complexes and SPLPs, SPLPs exerted superior efficacy and minimal toxicity at a 175 μg plasmid dose. In contrast, significant toxicity was observed for doses above 20 μg for liposome complexes following intravenous administration in mice [27]. Altogether, these results show the potential of SPLPs as a promising delivery platform for gene therapy. However, their challenging detergent dialysis formulation technique raised concerns over their manufacturability and scalability. This led to the development of the ethanol injection method wherein lipids dissolved in ethanol and pDNA in the acidic buffer are mixed and further diluted in an aqueous solution, leading to the spontaneous formation of LNPs [28]. Currently, LNPs are formulated using various methods; T-junction mixing, microfluidic hydrodynamic focusing (MHF), and staggered herringbone mixing (SHM) that contains ethanol as a phase to solubilize the lipids [29].

**Figure 2:**
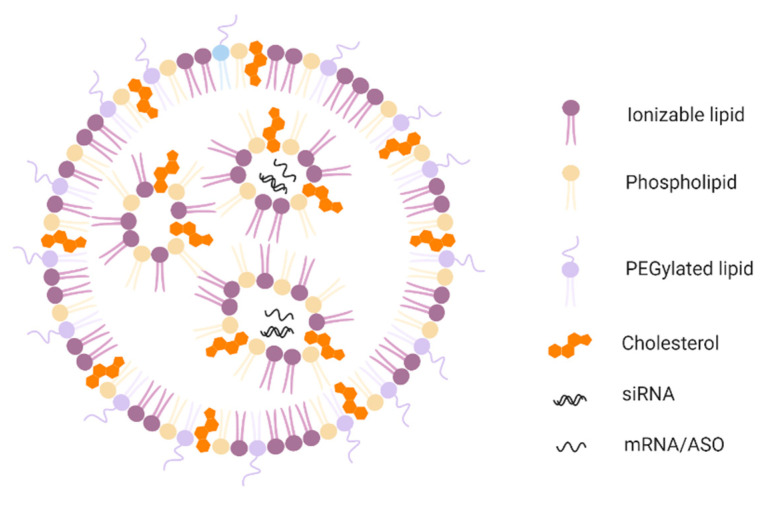
Structure of lipid nanoparticles (LNPs) comprising of all the components. An overall structural arrangement of lipid components used to formulate LNPs is shown. LNPs comprise of 4 main lipid components namely, ionizable lipids (pink), phospholipids (light yellow), polyethylene glycol (PEG)ylated lipids (light blue), and cholesterol (orange) encapsulating nucleic acid cargo such as double-stranded siRNA or single-stranded mRNA or antisense oligonucleotide (ASO).

**Figure 3:**
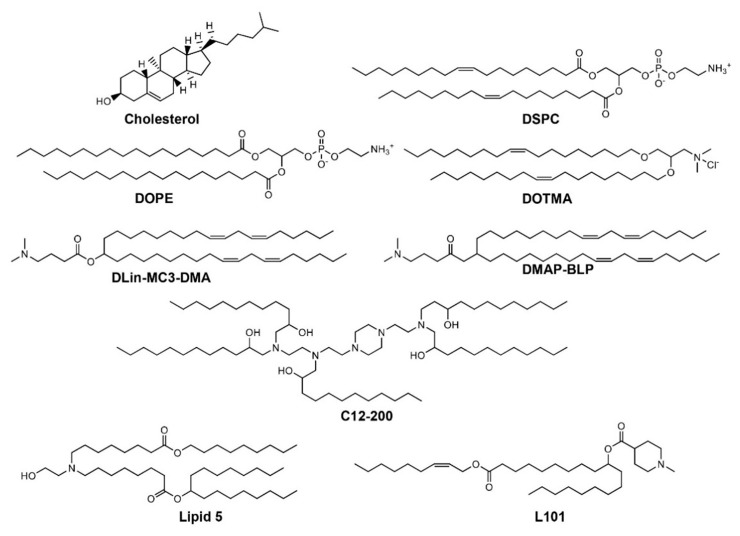
Chemical structures of cationic and neutral lipids for the preparation of LNPs for nucleic acid delivery. LNPs are formulated using cationic lipids that have an ionizable cationic amino head group and neutral helper lipids. Cationic lipids play an important part as they are essential for the stability and encapsulation of nucleic acids, whereas helper lipids tend to stabilize the lipid bilayers. DOTMA (N-[1-(2,3-dioleyloxy)propyl]-N,N,N-trimethylammonium chloride), DLin-MC3-DMA ([6Z,9Z,28Z,31Z]-heptatriacont-6,9,28,31-tetraene-19-yl 4-(dimethylamino)butanoate, C12-200 ((1,1′-((2-(4-(2-((2-(bis(2-hydroxydodecyl)amino)ethyl)n(2-hydroxydodecyl)amino)ethyl)piperazin-1-yl)ethyl)azanediyl)bis(dodecan-2-ol)) and heptadecan-9-yl 8-((2-hydroxyethyl)(8-(nonyloxy)-8-oxooctyl)amino)octanoate (Lipid 5), 3-(dimethylamino)propyl(12Z,15Z)-3-[(9Z,12Z)-octadeca-9,12-dien-1-yl]henicosa-12,15-dienoate (DMAP-BLP), (2Z)-non-2-en-1-yl 10-[(Z)-(1-methylpiperidin-4-yl)carbonyloxy]nonadecanoate (L101) are all cationic ionizable lipids. Cholesterol, 1,2-dioleoyl-sn-glycero-3-phosphoethanolamine (DOPE), and 1,2-distearoyl-sn-glycero-3-phosphocholine (DSPC) are neutral helper lipids.

Optimal amounts of cationic lipids are required for high encapsulation efficiency that can lead to an increase in cationic surface charge and subsequently cause toxicity. Hence, ionizable lipids containing an amino head group with an acid dissociation constant (pKa) below 7 are employed [30,31]. Low pKa allows ionizable lipids to be positively charged at acidic pH (<6.0) and neutral at physiological pH (7.4), which results in high encapsulation efficiencies for nucleic acids at acidic pH. Ionizable lipids and other helper lipids also interact with negatively charged membranes of the endosome that results in their membrane disruption and the release of nucleic acids [32]. Thus, ionizable amino lipids, along with helper lipids, comprise the lipid components of LNP formulations (Figure 2). Figure 3 depicts chemical structures of lipid components for the LNPs and liposome formulation along with recently developed novel ionizable amino lipids.

### 2.1. Messenger RNA (mRNA) Delivery by Lipid Based Vectors

The first demonstration of mRNA therapy was performed in 1992 where the intrahypothalamic delivery of vasopressin mRNA led to the temporary reversal of diabetes in rats for 5 days [33]. mRNA-based therapy has excellent potential as it requires only cytosolic delivery for efficacy without the risk of mutagenesis, as it cannot integrate into the host genome. However, mRNA delivery to target cells and tissues remains a significant challenge. With the recent progress on LNPs to deliver short interfering RNAs (siRNA), they have also been explored for mRNA delivery. However, to achieve the optimum in vivo efficacy, it is necessary to evaluate the translation of mRNA into protein in conjunction with its route of delivery. Pardia et al. established that LNPs can deliver luciferase mRNA and also compared their efficiency to translate into luciferase protein in a comprehensive in vivo study [34]. A molar ratio of DLin-MC3-DMA:PC:CHO:PEG-lipid (50:10:38.5:1.5) was used to encapsulate luciferase mRNA. After systemic delivery, protein levels were detected even after 6–10 days. The highest mRNA translation and amount of luciferase protein were observed after intravenous administration of 1–5 μg doses at 4 h, with levels reaching baseline expression on day 3.

In another study, novel lipid libraries were generated for erythropoietin (EPO) mRNA delivery. The formulation components included C12-200 (1,1′-((2-(4-(2-((2-(bis(2-hydroxydodecyl) amino) ethyl)n(2-hydroxydodecyl) amino) ethyl) piperazin-1-yl) ethyl) azanediyl) bis(dodecan-2-ol) as an ionizable lipid, 1,2-distearoyl-sn-glycero-3-phosphoethanolamine (DOPE) as a helper lipid, cholesterol (CHO), and C14-PEG2000 as a lipid-anchor PEG. Using a design of experiment (DOE) approach, different libraries of varying C12-200:mRNA weight ratio, molar compositions of lipid components, and phospholipids were tested. The optimized C35 formulation contained C12-200:DOPE:CHO:C14-PEG2000 (35:16:46.5:2.5) as lipid components with a C12-200:mRNA weight ratio of 10:1. The C35 formulation displayed a sevenfold increase in EPO protein expression as compared to the conventional formulation. Further, LNPs were tested to achieve the highest serum EPO levels after systemic delivery in mice. The optimized C35 formulation demonstrated threefold increase in EPO-based luciferase expression in the liver as compared to control groups [35].

Similarly, LNPs have been used for mRNA delivery to treat genetic diseases. Friedreich’s ataxia (FRDA) is a rare autosomal recessive neurodegenerative disease that leads to impaired motor functions. Reduced levels of frataxin (FXN), an essential protein found in sensory neurons, is associated with Friedreich’s ataxia patients. This leads to the degeneration of nerve tissue in the spinal cord and progression of the pathological disease state. Nabhan et al. delivered LNPs containing human FXN mRNA to supplement FXN protein and to specifically target dorsal root ganglia, which are most affected in FRDA. A molar ratio of DLin-MC3-DMA:DSPC:CHO:DMG-PEG2000 (55:10:32.5:2.5) was used to formulate LNPs. FXN mRNA delivered intravenously in mice yielded efficient FXN mRNA translation in the liver. Further, the intrathecal administration of LNPs produced optimal levels of human FXN in dorsal root ganglia as compared to the control group [36].

Although LNPs are good candidates for mRNA delivery, there are still concerns regarding their efficiency and safety profile from a clinical standpoint. Currently, DLin-MC3-DMA, an amino lipid, has shown great promise for mRNA delivery in clinical trials. DLin-MC3-DMA belongs to the class of ionizable lipids that constitute a significant component of LNP formulation for nucleic acid delivery [22]. Moderna Therapeutics has generated a novel series of amino lipids for LNP formulations for mRNA delivery and compared their pharmacokinetic and toxicity profile with DLin-MC3-DMA LNPs. A detailed exploration of the structure activity relationships of amino lipids have displayed an approximately threefold higher expression of luciferase mRNA delivered by LNPs based on lipid 5 (heptadecan-9-yl 8-((2-hydroxyethyl) (8-(nonyloxy)-8-oxooctyl) amino) octanoate) as an ionizable lipid as compared to DLin-MC3-DMA-based LNPs in mice. LNPs made from lipid 5 also showed higher liver clearance during in vivo studies. Further studies were performed in non-human primates (Cynomolgus monkeys), where lipid 5 and DLin-MC3-DMA-based LNPs encapsulating human EPO mRNA and anti-human immunoglobulin G (IgG) influenza A antibody mRNAs (0.01 mg/kg via IV fusion) were injected systemically. It was noted that lipid 5-based LNPs showed fivefold higher mRNA expression (for both EPO and anti-human IgG influenza A) as compared to DLin-MC3-DMA LNPs. Lipid 5-based LNPs also demonstrated superior pharmacokinetic profile with decreased liver accumulation at clinically relevant doses (1–2 mg/kg dose per week over 5 weeks) in both rats and cynomolgus monkeys [37]. Thus, ionizable lipids as components of LNPs have shown great potential for mRNA delivery.

LNPs have also been explored for the delivery of mRNA-based vaccines. There has been a considerable interest in developing a vaccine for the ZIKA virus [38,39,40]. Richner et al. evaluated the LNP-based formulation to deliver mRNA of ZIKA viral proteins [41]. A modified mRNA was chosen that can translate precursor membrane (prM) and Envelope (E) surface protein genes from the Asian ZIKA viral strain along with the signal sequence of human IgE (IgE_sig_-prM-E mRNA). 3-(dimethylamino)propyl(12Z,15Z)-3-[(9Z,12Z)-octadeca-9,12-dien-1-yl]henicosa-12,15-dienoate (DMAP-BLP) based lipid was used in the LNP formulation. A molar ratio of DMAP-BLP:DSPC:CHO:PEG-lipid (50:10:38.5:1.5) was used to generate LNPs containing IgE_sig_-prM-E mRNA. The intramuscular administration of LNPs with booster doses at 8 and 14 weeks in immunocompetent mice led to increased serum neutralizing titers (1/10,000). Immunized mice also displayed superior survival post-infection with the ZIKA virus as compared to the control group. Furthermore, modified vaccines based on Japanese encephalitis viral IgE sequence (JEV_sig_-prM-E LNPs) administered at a dose of 2–10 μg displayed neutralizing titers of approximately 1/100,000 with minimal viremia.

### 2.2. siRNA Delivery by Lipid Based Vectors

One of the earliest discoveries of RNA interference (RNAi) is a biological process was noted in *Caenorhabditis elegans*, where RNA controls the gene expression by targeting mRNA strands [42,43]. Further studies led to the identification of small interference RNA (siRNA) having shorter sequences of about 21–23 nucleotides in length, which are generated from the cleavage of longer double-stranded (ds) RNA and are capable of silencing various genes [44]. In RNAi, a series of silencing protein complexes bind to siRNA forming an RNA-induced silencing complex (RISC), which cleaves the complementary mRNA transcript, leading to gene silencing and suppression [45,46,47]. siRNA delivery has been challenging due to inadequate in vivo stability and cellular uptake issues. Patisiran (Onpattro^®^) is the first LNP–siRNA formulation to have been approved by the U.S. Food and Drug administration (FDA) for the treatment of hereditary transthyretin (hTTR) amyloidosis. hTTR amyloidosis is a hereditary disorder that results in neuropathy as well as cardiomyopathy. TTR is an essential protein that is responsible for the transportation of thyroxine and retinol. TTR protein contains β strands that become folded due to single point mutations in the TTR gene and which results in deposits of insoluble TTR protein, causing amyloidosis [48,49]. Patisiran treatment improved the polyneuropathy scores in patients and led to the reversal of disease progression [50]. Patisiran formulation contains ds siRNA (ALN-18328), wherein each strand has 21 nucleotides. Further, siRNA is encapsulated in LNPs composed of DLin-MC3-DMA as the ionizable lipid, DSPC and CHO as helper lipids, and PEG2000-C-DMG [α-(3′-{[1C-di(myristyloxy)proponoxy] carbonylamino}propyl)-ω-methoxy, polyoxyethylene]) as a lipid PEG anchor [51]. Patisiran formulations target liver hepatocytes, which is the primary site for the synthesis of TTR protein. Post IV infusion of Patisiran in 29 patients, there was a 20–30% reduction in TTR protein levels 24 h after the first dose, followed by an 85% reduction after the second dose, and maximum knockdown of 96% of TTR was observed after the third dose at 0.3 mg/kg [52].

In another promising finding, LNPs have been shown to co-deliver both mRNA and siRNA with optimal efficacy, wherein the other RNA acts as a helper. The LNP formulation consisted of a molar ratio of ionizable lipid:DSPC:DOPE:CHO:C14-PEG2000 (38.8:3.6:10.9:44.5:2.25) with a lipid to RNA weight ratio of 8.75:1. siRNA targeting Factor VII gene and mRNA for luciferase protein were encapsulated in LNPs. siRNA silencing was increased twofold, whereas luciferase expression due to mRNA delivery increased three times following tail vein injection in mice. The substitution of either RNA by a negatively charged polymer (polystyrene sulfonate) as a helper RNA did not affect the efficacy [53].

LNPs must show a superior pharmacokinetic profile with reduced toxicity. Therefore, the careful selection of ionizable lipids is critical for the success of LNP formulations. This led to the development of biodegradable LNPs, which show good hepatic clearance along with functionality to achieve superior efficacy. A biodegradable ionizable lipid (2Z)-non-2-en-1-yl 10-[(Z)-(1-methylpiperidin-4-yl)carbonyloxy]nonadecanoate (L101) was used to formulate biodegradable and effective LNP formulations. Biodegradable LNPs were made with a molar ratio of L101:DSPC:CHO:PEG-DMG (60:8.5:30:1.5) encapsulating an siRNA targeting proprotein convertase subtilisin/kexin type 9 (PCSK9). PCSK9 is a protein majorly synthesized in the liver that binds to the LDL receptor and regulates the transport of lower-density lipoprotein (LDL) particles. Irreversible binding of the PCSK9 protein to the LDR receptor leads to their loss of function to internalize the LDL particles. This process leads to elevated LDL levels in the plasma, followed by an increasing risk for cardiovascular disease [54,55]. PCSK9-targeted siRNA LNPs displayed high efficacy with more than 90% protein silencing following IV administration in non-human primates. This particular formulation also showed higher hepatic clearance and therefore exhibited a superior safety profile [56].

### 2.3. Antisense Oligonucleotide Delivery by Lipid Based Vectors

Antisense oligonucleotides (ASOs) are synthetic nucleic acids that hybridize and bind to mRNA by Watson–Crick base pairing and lead to the formation of an ASO–mRNA heteroduplex. Antisense strategy was first reported by Zamecnik et al., where they explored a 13 nucleotide sequence to target 35S RNA of Rous sarcoma virus, which led to the inhibition of viral replication [57]. Although ASOs possess enormous potential for gene therapy, certain challenges need to be addressed to achieve high therapeutic efficacy as well as clinical application. ASOs are often susceptible to rapid degradation by enzyme nucleases, display poor cellular uptake and pharmacokinetic profile, as well as moderate efficacy in vivo [58,59,60]. This lead to the development of a new generation of ASOs: phosphorothioates (PS), locked nucleic acids (LNA), morpholinos, and peptide nucleic acids (PNA) based on either chemical modifications in phosphodiester linkage or in the ribose sugar moiety to overcome aforementioned challenges [61]. More recently, Inotersen (Tegsedi^TM^), a second generation 2′-O-methoxyethyl modified ASO, received global approval for the treatment of hTTR in 2018 [62].

PS are synthetic nucleic acids that possess a modified chemical structure wherein the oxygen atom is replaced with sulfur in the phosphodiester linkage, which prevents their enzymatic degradation by nucleases and improves the bioavailability [60]. LNA display a pre-organized helical conformation due to O-methyl bridge at the 2′ and 4′ carbon in the ribose sugar. This bridge provides an ideal conformation and increases the binding affinity of LNAs with complementary target sequence [63]. Morpholinos show the presence of morpholine and phosphorodiamidate instead of ribose sugar and phosphodiester linkage, respectively [64]. PNA contain chiral N-(2-aminoethyl)-glycine units as a backbone [65]. They demonstrate higher binding affinity to complementary DNA and RNA sequences due to their neutral charge [2].

Furthermore, the encapsulation of ASOs, as mentioned above in carrier systems, grants them further protection from nucleases and improves their cellular uptake and biodistribution. In general, ASOs demonstrate their efficacy by one of the following mechanisms: (1) recruitment of RNase H at an mRNA–ASO heteroduplex site, which results in the cleavage of target mRNA [66], (2) the regulation of mRNA splicing where ASOs binds to an aberrant pre-mRNA splicing site and restore protein translation [67,68], and (3) ASOs bind to mRNA and result in steric hindrance between mRNA and the ribosome [63,66,69,70,71].

However, the therapeutic delivery of ASOs always exerts an enormous challenge for their broader clinical application. LNPs have made promising strides in the delivery of therapeutically active ASOs. Yang et al. developed an LNP-based formulation for ASO delivery [72]. They generated a library of bioreducible lipids and chose the best three candidates for in vitro testing of PS and 2′ modification-based ASOs in HEK cells. Lipid 306-O12B-3 was chosen to formulate LNPs at a weight ratio of lipid/CHO/DOPE/DSPE-PEG2000 (16/4/1/1) to encapsulate ASOs targeting PCSK9 mRNA. After the systemic delivery of ASOs in mice, significant PCSK9 mRNA silencing was noted with minimal toxicity at a dose of 5 mg/kg.

In conjunction with the optimal delivery strategy, ASOs have shown tremendous potential for targeting oncogenic mRNA for cancer therapy [73]. Cheng et al. delivered ASO G3139 (Oblimersen) using LNP-based formulations. LNPs were formulated using the molar ratio of DOTAP:eggPC:CHO:Tween 80 (25:50:20:5) to encapsulate G3139 ASOs to target *BCL-2*, which is an anti-apoptotic gene overexpressed in a variety of cancers. An initial screen of LNP formulation was performed on A549 (epithelial adenocarcinoma) cell lines. LNPs delivered G3139 efficiently and downregulated *BCL-2* expression to approximately 40% and approximately 83% for mRNA and protein, respectively. G3139–GAP LNPs, in combination with Paclitaxel, were tested in vivo in xenograft mice. Systemic treatment with G3139-GAP LNPs yielded the highest median survival time (approximately 57.3 days) at a dose of 5 mg/kg in mice along with highest reduction in *BCL-2* expression in tumors as confirmed by immunohistochemistry staining compared to control groups [74].

## 3. Cationic Polymers

**Figure 4:**
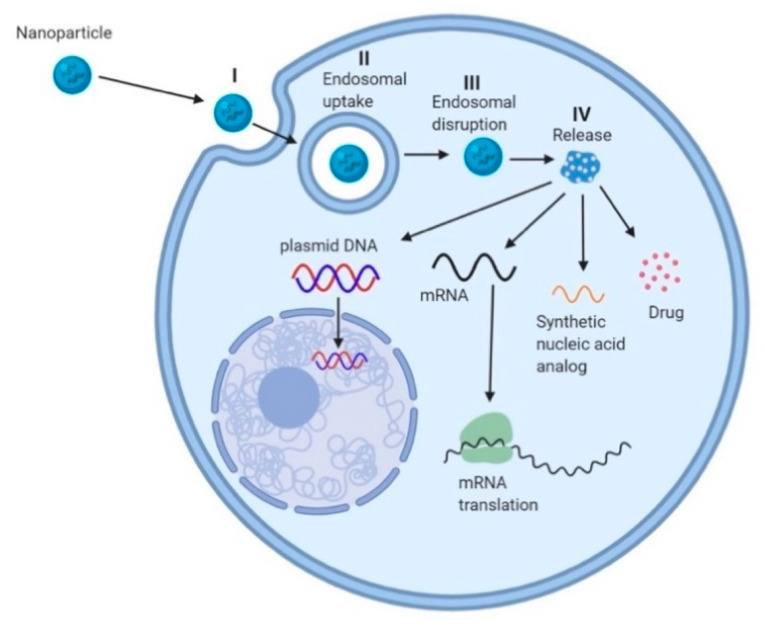
Schematic showing cellular uptake of cationic polymeric nanoparticles by endocytosis and delivery by endosomal disruption. Mechanism for delivery of nucleic acids follows 4 crucial steps. STEP I is the initialization of cellular uptake of polymeric nanoparticles via endocytosis. Cationic polymers having a positive charge helps in improving the cellular uptake, as it facilitates interaction with the negatively charged cellular membrane. STEP II is the endosomal uptake of nanoparticles, which is the fate for any foreign particles entering the cell. STEP III is endosomal disruption, which leads to release of the nanoparticles. Cationic polymers facilitate the disruption of endosomes as they act as proton quenchers, owing to their positive charge. This facilitated endosomal disruption aided by cationic species is called the ‘Proton sponge effect’. STEP IV is release of the encapsulant into the cytoplasm following degradation of the polymer. The encapsulant has now access to cellular machinery to show efficacy.

**Figure 5:**
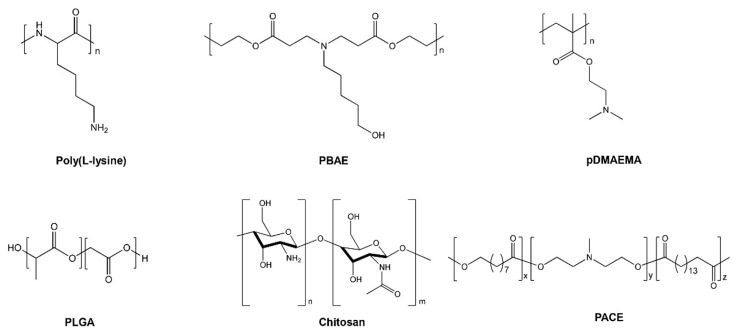
Chemical structures of poly(L-lysine), poly(β-amino ester) (PBAE), poly[(2-dimethylamino) ethyl methacrylate] (pDMAEMA), poly(lactic-co-glycolic acid) (PLGA), chitosan, and poly(amino-co-ester) (PACE).

**Figure 6:**
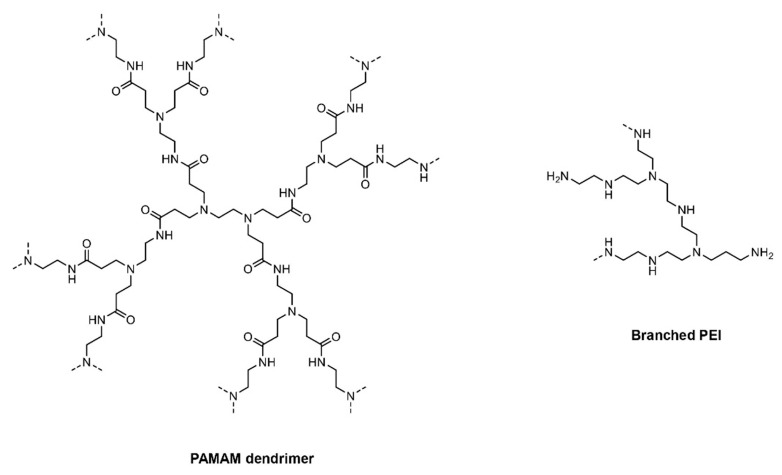
Chemical structures of polyamidoamine (PAMAM) dendrimers and poly-ethylenimine (PEI).

Carrier systems containing cationic polymers have an added advantage of formulating smaller uniform particle size, which leads to improved transfection efficiency. Cationic polymers tend to condense and pack the negatively charged nucleic acids [75]. Poly-L-lysine (PLL) was the first cationic polymer investigated for DNA transfection [76]. Further, Boussif et al. synthesized and tested poly-ethylenimine (PEI), which is a novel branched cationic polymer having the highest cationic charge density [75]. PEI consists of a highly branched network that is capable of undergoing protonation due to its charged amino group [75]. The higher transfection efficiency of PEI is attributed to the buffering capacity of multiple amino groups on PEI, which can quench protons pumped by the vesicular ATPase proton pump present on the endosomes [77]. This ‘proton-sponge effect’ of PEI leads to an influx of chloride ions and water in the endosome, which eventually leads to osmotic swelling and endosomal disruption [75]. Vermeulen et al. explored essential factors that govern the endosomal escape of PEI formulations in different cell lines. Using JetPEI polyplexes utilizing plasmid DNA, endosomal compartment size, and leakiness were reported as the factors to facilitate higher endosomal escape and transfection [78]. Recently, Wojnilowicz et al. studied different polyplexes for the delivery of siRNAs and tracked the trafficking of siRNAs following internalization in prostate cancer cells (PC3 cells) using stochastic optical reconstruction microscopy (STORM). The snapshots from STORM indicated that only rigid and highly branched polyplexes such as glycoplexes, PEI, and solid silica nanoparticles displayed a proton sponge effect and thereby endosomal disruption, suggesting them to be important pre-requisites for facilitating endosomal escape [79]. Figure 4 shows the fate of cationic polymeric nanoparticles as they undergo cellular uptake and the delivery of cargo by endosomal disruption. However, PEI-based formulations exert cytotoxicity because of binding to serum proteins and erythrocytes due to their high positive charge, thereby causing plasma membrane disruption [14,80,81]. Moreover, it has been established that cell lines treated with PEI polymers show autophagy, necrosis, and apoptosis [82]. Hence, to resolve the aforementioned issues, next-generation cationic-based polymers—poly[(2-dimethylamino) ethyl methacrylate] (pDMAEMA), polyamidoamine (PAMAM) dendrimers, and biodegradable poly(β-amino ester) (PBAE) polymers—were developed [5]. Due to tertiary amine end groups, pDMAEMA and PBAE also aid in endosomal escape and demonstrate superior transfection efficiency. Although PBAE shows less toxicity as compared to PEI, still caution needs to be exercised considering their surface charge density [83]. Hence, to increase the transfection efficiency and decrease the non-specific binding, novel, new generation poly(amino-co-ester) (PACE)-based polymers were developed and optimized for nucleic acid delivery [84]. Figure 5 and Figure 6 depict the chemical structures of cationic polymers commonly used for the delivery of nucleic acids.

The primary foundation of PACE polymers is based on the presence of branched amino groups to aid in endosomal disruption and have a cleavable ester moiety that can readily hydrolyze in biological conditions [85]. PACE-based cationic polymers show minimal cytotoxicity as compared to other classes of cationic polymers. Higher molecular weight (MW) PACE polymers were synthesized using more hydrophobic moieties, thereby reducing the cationic charge and reducing systemic cellular toxicity [86]. High MW PACE possesses superior transfection efficiency because of the significant condensation of DNA and the formation of stable DNA complexes [87].

### 3.1. mRNA Delivery by Cationic Polymeric Vectors

Poly (β-amino esters) are biodegradable polymers that show higher transfection efficiency owing to endosomal escape and show lower toxicity as compared to PEI cationic polymers. However, to reduce the overall surface charge of PBAEs, hyperbranched PBAEs (hPBAEs) were developed. hPBAEs of hDD90-118 were made to encapsulate luciferase mRNA and delivered in mice by inhalation. hPBAEs-treated mice showed optimal luciferase protein levels (101.2 ng/g) after 24 h of inhalation [88].

Similarly, poly(amino-co-ester) (PACE) has been extensively used for the mRNA delivery. In general, polymeric materials for gene delivery are synthesized by the “bottom–up” approach. However, the “bottom–up” approach can be difficult and possesses numerous challenges, especially on a commercial scale-up level. Saltzman lab has developed next-generation actuated PACE (aPACE) by the “top–down” approach [89], which involves simultaneously changing the functional end group compositions and molecular weight (MW) of the polymer. The approach mentioned above involves an actuation process that leads to mild temperature and air exposure to initiate the hydrolysis of the ester backbones to generate different MW PACE polymers containing generations of –COOH and –OH terminal end groups. Overall, it was demonstrated that MW of 5 kDa is sufficient to enable the complexation of aPACE with the mRNA and their subsequent optimal release. aPACE possess superior mRNA delivery (with up to a 10^6^ fold increase) properties as compared to regular PACE in vitro. In addition, aPACE efficiently delivered mRNA coding for erythropoietin (EPO) in vivo and produced high levels in the blood for up to 48 h without inducing any systemic toxicity. This study explains the overall versatility of PACE polymers wherein their chemistry could be optimized for a wide range of mRNA delivery-based therapeutic applications. Further, a library of PACE polymers with different end groups was tested to assess their superior endosomal escape and transfection efficiency. It was noted that mRNA encapsulation efficiency and endosomal escape plays an essential role in determining the transfection efficiency of PACE polymers [90]. aPACE constitutes a new delivery strategy for mRNA-based treatments that provides safe and potent protein production.

### 3.2. siRNA Delivery by Cationic Polymeric Vectors

Tissue transplantation is a vital therapeutic area that has been explored for treating various devastating diseases. Though successful, still, the host adaptive immunity to the grafted tissues is a major challenge for the transplantation-based therapies. Human endothelial cells containing class II transactivator (CIITA) major histocompatibility complex (MHC) molecules regulate immune activation and therefore are considered as important components of the host rejection response. Hence, the treatment of graft endothelial cells with siRNA targeting CIITA would reduce the expression of MHC class II molecules and thereby lead to immunosuppression with better allograft acceptability. To achieve this, PACE-based formulations were explored as a carrier system for siRNA targeting of CIITA. A series of PACE polymers were synthesized based on increasing the composition of 15-pentadecanolide (PDL) to achieve higher hydrophobicity, which generated solid nanoparticles and were tested in the HUVEC (human umbilical vein endothelial cells) cell line for cytotoxicity. PACE 90 (containing 90% PDL content) nanoparticles were chosen for further ex vivo study as they displayed minimal cytotoxicity. It has been demonstrated that the single ex vivo transfection of PACE containing siRNA weakens the MHC class II expression in human arteries for approximately 4 to 6 weeks after transplantation into immunodeficient mice. This study concludes the advantage of PACE polymers to modulate the release and excellent encapsulation efficiency of siRNA [91].

siRNA delivery has been limited due to challenges such as poor in vivo stability and membrane penetration. The upregulation of Nogo-B protein is seen as a biomarker for hepatic fibrosis and alcoholic liver disease. The subsequent knockdown of Nogo-B protein would thereby lead to healthy liver function, suggesting a suitable starting point for their siRNA-based therapy. A range of PACE polymers were synthesized to achieve the maximum transfection of pLucDNA (plasmid Luciferase DNA), and cytotoxicity was evaluated in HEK293 and HUVEC cell lines followed by in vivo studies. PACE 70 (solid PACE nanoparticles made from 70% 15-pentadecanolide (PDL)) showed increased uptake in Kupffer cells, liver sinusoidal endothelial cells, and hepatic stellate cells when administered systemically. Subsequently, PACE 70 siRNA nanoparticles injected in spleen showed >60% knockdown of Nogo-B protein in the liver. The direct injection of nanoparticles in the spleen was conducted as it displays the same biodistribution of nanoparticles in the liver when compared with intravenous administration [92].

### 3.3. Antisense Oligonucleotide Delivery by Cationic Polymeric Vectors

Cationic carriers have been extensively deployed for the delivery of ASOs. Chitosan is a naturally occurring cationic biodegradable polymer used for the delivery of nucleic acids. It was demonstrated that chitosan-coated poly(lactic-co-glycolic acid) (PLGA) nanoparticles possess positive surface charge that assist to condense nucleic acid cargo and display their burst release kinetics at pH 7 with an acceptable safety profile [93]. In this study, chitosan-coated PLGA nanoparticles were used to deliver 2′-O-methyl-RNA (OMR) to target human telomerase RNA for the treatment of lung cancer. Human telomerase is a ribonucleoprotein that initiates the addition of TTAGGG repeat units onto the end of chromosomes [94]. Although telomerase activity is associated with aging and cell proliferation, the overexpression of telomerase leads to cancer [95]. 2′-O-methyl-RNA is a second generation oligonucleotide that contains a phosphorothioate backbone and substantially inhibits human telomerase activity [96]. Chitosan-coated PLGA nanoparticles showed higher cellular uptake of OMR-based ASOs in A549 cell line as compared to PLGA nanoparticles [97].

MicroRNAs (miRNA) were first identified in 1993 in *Caenorhabditis elegans* [98]. miRNA belong to a class of non-coding RNA (ncRNA) that controls the gene expression by targeting mRNAs [99,100,101]. It has been well established that the abnormal expression of miRNAs leads to cancers [102]. In particular, miR-21 is known to be upregulated in glioblastoma (GBM). Hence, anti-miR-21 based ASOs were loaded into PACE polymers and explored for GBM therapy [103]. Furthermore, apolipoprotein E was conjugated with PACE nanoparticles to improve its stability as well as brain penetration properties. Anti-miR-21 PACE nanoparticles showed improved uptake in RG2 cells and optimal miR-21 knockdown in U87 cells with increased PTEN upregulation, which is a downstream target of miR-21. Furthermore, anti-miR-21 PACE nanoparticles, in combination with temozolomide, showed a significant knockdown of miR-21 and improved survival in rats with U87 intracranial tumors when administered by convection-enhanced delivery [103]. This study highlights the robustness of PACE polymers to deliver and release anti-miRs in combination with other chemotherapeutic agents for improved therapy for GBM and for intra-tumoral delivery.

## 4. Conjugate Delivery Systems

Surface modifications are often employed on gene delivery vectors to ensure cell/site-specific targeting and to improve the stability of nucleic acid cargo [104]. The cationic polymers when used to coat negatively charged nanoparticles increase their cytosolic delivery. Lee et al. demonstrated that PBAE-coated gold nanoparticles conjugated with siRNA targeting luciferase expression displayed >90% luciferase gene silencing in HeLa (cervical cancer cell line) cells as compared to control group [105].

Antibody-based therapy has seen a major revolution as polyspecific monoclonal antibodies are capable of targeting multiple tumor antigens with specificity without affecting normal bystander cells [106]. More than 60 antibody–drug conjugates are currently undergoing clinical trials [107], and 6 antibody–drug conjugates have received FDA approval for cancer therapy [108]. Similarly, antibody-conjugated nanoparticles containing nucleic acids have been explored for site-specific targeting [109]. Recently, Okamoto et al. established anti-heparin binding epidermal growth factor (anti-HB-EGF) antibody-conjugated LNPs containing siRNA targeting polo-kinase 1 (PLK-1) for triple negative breast cancer therapy. PLK-1 is associated with tumor cell growth and division for triple negative breast cancer. Since triple negative breast cancer tumors display an overexpression of HB-EGF, the conjugation of anti-HB-EGF antibody with LNPs (αHB-EGF LNP-siRNA) led to its increased tumor accumulation in MDA-MB-231 (triple negative breast cancer cell line) xenografted mice as compared to control LNPs after systemic administration. Moreover, the treatment of αHB-EGF LNP-siPLK-1 in mice yielded lower PLK-1 protein levels and tumor growth as compared to control groups [110]. This study highlights the use of antibody-conjugated non-viral carriers being able to deliver siRNA for the treatment of triple negative breast cancer.

In addition to the coating of delivery systems, direct conjugation with chemical scaffolds such as cholesterol to synthetic nucleic acid mimics such as siRNAs is also performed to increase their specificity as well as decrease the enzymatic degradation after in vivo delivery [111,112]. Covalently linked ligands are designed to facilitate receptor uptake and the delivery of nucleic acids to the target tissue. N-acetylgalactosamine (GalNAc) conjugate (also known as enhanced stabilization chemistry) and dynamic polyconjugate (DPC)-based technologies are the most explored conjugate delivery systems and have been used clinically for the treatment of liver diseases by Alnylam Pharmaceuticals [113] and Arrowhead Research Corporation [114,115] for siRNA delivery.

The GalNAc approach is widely used for siRNAs targeting liver hepatocytes. The GalNAc site specifically targets asialoglycoprotein receptor (ASGPR), which is overly expressed in hepatocytes [116]. Givosiran (Givlaari^TM^), the second siRNA drug approved by the FDA for the treatment of acute intermittent porphyria (AIP) developed by Alnylam Pharmaceuticals contains three GalNAc residues covalently linked to the siRNA targeting delta-aminolevulinic acid synthetase 1 [117]. A number of GalNAc-conjugated siRNA drug candidates—Cemdisiran [118,119], Lumisiran [120], Revusiran [121], Fitusiran [122,123] and Inclisiran [124,125,126]—have entered in clinical trials. Similarly, IONIS-ANGPTL3-LRx [127], GSK3389404/IONIS-HBV-LRx [128], IONIS-FB-LRx [129], and IONIS-PKK-LRx [130] are GalNAc-conjugated antisense oligonucleotides currently in clinical trials.

On the other hand, the first-generation DPC technology utilized an endosomolytic polymer PBAVE (poly(butyl amino vinyl ether) where PEG-GalNAc (PEG-N-acetylgalactosamine) is conjugated on one end and siRNA cargo is conjugated at other end by reversible disulfide linkage. In DPC technology, PEG chains provide enzymatic stability during systemic circulation and GalNAc residues ensure hepatocyte uptake through the asialoglycoprotein receptor. In an acidic environment, the PBAVE polymer leads to endosomal disruption and the release of siRNA in the cytoplasm [131]. The second generation of DPC relied on the co-injection of cholesterol-siRNA (Chol-siRNA) and PBAVE polymer covalently linked with PEG and GalNAc [132]. The co-injection of Chol-siRNA targeting ApoE protein and PBAVE polymer led to a 500-fold improvement in efficacy as compared to the administration of Chol-siRNA alone following systemic administration in mice. The above-mentioned technology was further optimized by Arrowhead and utilized for the delivery of ARC-520, which is a lead candidate for the treatment of hepatitis B virus (HBV) infection wherein 2 Chol-siRNAs were used along with melittin peptide as the osomolytic polymer instead of PBAVE [114].

## 5. Genome Editing

Recent advances in genome editing technology possess a vast potential to treat genetic disorders by correcting mutated genes by using cells’ own repair and recombination machinery [133]. Major tools in nuclease-based genome editing research includes zinc-finger nucleases (ZFN), transcription activator-like effector nucleases (TALENs), and more recently clustered regularly interspaced short palindromic repeats/Cas9 (CRISPR/Cas9). All aforementioned technologies rely on the propensity to generate double-strand breaks (DSB) at the target chromosomal DNA sequence and initiate cells’ endogenous repair mechanisms of non-homologous end joining (NHEJ) or homology-directed repair (HDR) [134,135]. ZFNs contain engineered DNA binding domain designed to identify target DNA sequence fused with DNA cleavage domain from *Fok1* endonuclease [136]. Similarly, TALENs also employ a customizable DNA binding domain consisting of transcription activator-like effectors (TALEs) to bind to the DNA target fused with non-specific DNA cleavage domain from the *Fok1* endonuclease [137]. On the other hand, the CRISPR/Cas9 system relies on synthetic single guide RNA (sgRNA) to hybridize with a target DNA sequence, following which Cas9 initiates DSB [138,139]. However, the in vivo delivery of either of above-mentioned gene editing modalities to target tissues has always been a major challenge and prevents their translation to the clinic.

Mahiney et al. employed chitosan-coated PLGA nanoparticles to deliver ZFN-mRNA with a donor template in surfactant protein B deficient mice by intrathecal administration. The delivery of ZFN-mRNA led to a correction of genes and improved survival for mice with low immune response [140]. Conway et al. used LNP-based formulation to deliver ZFN-mRNA targeting TTR (transthyretin) and PCSK9 gene in mice by systemic delivery. This led to 80% and 90% protein reduction levels respectively at 10-fold lower mRNA doses [141]. CRISPR/Cas9 genome editing, a novel delivery system developed by Sun et al., explored employing self-assembling nanoparticles comprising of DNA nanoclews (NCs), which are designed to contain sequences that are partially complimentary to sgRNA enabling the efficient loading of the sgRNA–Cas9 complex. To ensure endosomal escape, PEI coating was applied to DNA NC nanoparticles. DNA NC nanoparticles loaded with RNP (ribonucleoproteins such as Cas9) targeting the EGFP gene when administered intratumorally to U2OS.EGFP tumor xenografted mice led to a 25% reduction in EGFP gene expression in U2OS.EGFP tumor cells after 10 days [142]. Further, Jiang et al. developed lipid-like particles (LLN) to deliver Cas9 mRNA and sgRNA targeting the proprotein convertase subtilisin/kexin type 9 (PCSK9) gene for the treatment of hypercholesterolemia. LLN when administered in C57BL/6 mice via tail vein displayed high Cas9 protein levels 6 h post injection with a rapid decline at 12 h and reduced levels in PCSK9 protein as compared to control groups. The group also screened several sgRNAs and optimized a lead sgRNA (sgRNA B5) targeting hepatitis B viral covalently closed circular DNA. sgRNA B5 when administered post 6 h of Cas9 mRNA-LLN tail vein injection in C57BL/6 mice resulted in a significant reduction of liver hepatitis B surface antigens [143].

## 6. Conclusions

Overall, numerous non-viral based strategies have been employed to improve nucleic acid delivery for next-generation gene therapy. Cationic lipids and polymers have made significant progress in the delivery of various classes of nucleic acids for therapeutic purposes (Table 1). Likewise, numerous non-viral vectors are undergoing clinical trials for nucleic acid therapy (Table 2).

However, ligand-targeted delivery for cationic lipids and polymers still needs to be explored to increase their applicability at the clinical level. The pros and cons of lipid-based vectors and cationic polymeric vectors are summarized in Table 3. Nonetheless, with the advent of personalized medicine, non-viral vectors possess enormous potential to deliver therapeutically active drugs or drug candidates to an organ of choice and treat myriads of devastating diseases.

## Figures and Tables

**Table 1:** Brief summary of all the nucleic acid vectors.

SN	Nucleic Acid	Gene/Target	Vector	Disease/Condition	Route of Delivery	Animal/Cell Line Used	Ref.
1	mRNA	EPO	LNPs	-	IV	C57BL/6 mice	[35]
2	mRNA	Luciferase	LNPs	-	IP, IM, SC, IV, ID and ITr	BALB/c mice	[34]
3	mRNA	FXN	LNPs	Friedreich’s ataxia	ICV	BALB/c mice	[36]
4	mRNA	Luciferase and hEPO	LNPs	-	IV	CD-1 mice, rats, cynomolgus monkeys	[37]
5	mRNA	pRM and E	LNPs	Zika	IM	AG129 mice	[41]
6	siRNA and mRNA	Factor VII and luciferase	LNPs	-	IV	C57BL/6 mice	[53]
7	siRNA	PCSK9	LNPs	Elevated LDL-Cholesterol	IV	C57BL/6 mice and monkeys	[56]
8	mRNA	PCSK9	LNPs	Elevated LDL-Cholesterol	IV	BALB/c mice	[72]
9	G3139-GAP	Bcl-2	LNPs	Lung cancer	IV	BALB/c mice	[74]
10	mRNA	Luciferase	hPBAEs NPs	-	Inhalation	C57BL/6 mice	[88]
11	mRNA	EPO	PACE NPs	-	IV	BALB/c mice	[89]
12	siRNA	CIITA	PACE NPs	Tissue transplantation	Incubation	SCID/beige mice	[91]
13	siRNA	Nogo-B	PACE NPs	Hepatic fibrosis and alcoholic liver disease	Spleen	C57BL/6 mice	[92]
14	ASO	Human telomerase	Chitosan-coated PLGA NPs	Lung cancer	Cellular uptake	A549 cancer cell line	[97]
15	ASO	miR-21	PACE NPs	Glioblastoma	CED	Fischer 344 rats	[103]
16	siRNA	PLK-1	EGF antibody Anti-HB -LNPs	Triple negative breast cancer	IV	BALB/c mice	[110]
17	siRNA	ApoB	PBAVE NPs	-	IV	ICR mice	[132]
18	ZFN mRNA	SFTB	Chitosan-coated PLGA NPs	Lung disease	ITh	SP-B transgenic mice	[140]
19	ZFN mRNA	TTR and PCSK9	LNPs	Elevated LDL-Cholesterol and amyloidosis	IV	CD-1 mice	[141]
20	Cas9 and sgRNA	EGFP	PEI coated DNA nanoclew	Osteosarcoma	IT	Nude mice	[144]
21	Cas9 and sgRNA	PCSK 9 and HBV	LLNs	Elevated LDL-Cholesterol and Hepatitis B	IV	C57BL/6 mice	[142]

Abbreviations: EPO, erythropoietin; LNPs, lipid nanoparticles; FXN, frataxin; FVII, protein Factor VII; PCSK9, proprotein convertase subtilisin/kexin type 9; bcl-2, B-cell lymphoma 2; prM, pre-membrane protein; E, envelope protein; CIITA, class II transactivator; Nogo-B, Nogo-B gene, a part of Nogo/Reticulon-4B family; hPBAEs, hyper branched poly-β-amino esters; PACE, poly(amino-co-ester); PLGA, poly(lactic-co-glycolic acid); miR-21, microRNA-21; PLK-1, polo-kinase 1; ApoB, apolipoprotein; PBAVE; poly(butyl amino vinyl ether) polymer; PEI, polyethylenimine; TTR, transthyretin; EGFP, epidermal growth factor protein; SFTB, surfactant B gene encoding for surfactant B protein; sgRNA, single guide RNA; HBV, hepatitis B virus; IV, intravenous; IP, intraperitoneal; IM, intramuscular; SC, subcutaneous; ID, intradermal; ITr, intratracheal; ICV, intracereboventicular; IT, intratumoral; ITh, intrathecal; CED, convection enhanced delivery.

**Table 2:** Non-viral vectors currently in clinical trials.

SN	Nucleic Acid	Target	Vector	Disease	Route of Delivery	Clinical Trial	Status
1	mRNA	OX40L	LNPs	Solid tumors and lymphomas	IT	NCT03739931	Active
2	mRNA	OX40L	LNPs	Solid tumors, lymphomas and ovarian cancer	IT	NCT03323398	Active
3	mRNA	S-protein	LNPs	COVID-19	IM	NCT04283461	Active
4	mRNA	OTC	LNPs	OTC deficiency	IV	NCT03767270	Withdrawn
5	mRNA	prM and E	LNPs	Zika	IM	NCT04064905	Active
6	mRNA	Pentamer and T cell antigen	LNPs	CMV infection	IM	NCT03382405	Active
7	siRNA	MYC	LNPs	Hematological and solid tumors	IV	NCT02110563	Terminated
8	siRNA	MYC	LNPs	Hepatocellular carcinoma	IV	NCT02314052	Terminated
9	siRNA	HSP47	LNPs	Hepatic fibrosis	IV	NCT02227459	Completed
10	siRNA	PLK1	LNPs	Solid tumors	Hepatic IA	NCT01437007	Completed
11	saRNA	CEBPA	Liposomal NPs	Hepatocellular carcinoma	IV	NCT02716012	Active
12	siRNA	TGF-β1 and Cox-2	NPs	Hypertrophic scar	ID	NCT02956317	Unknown
13	siRNA	KRAS	PLGA matrix	Adeno-carcinoma	SI	NCT01676259	Recruiting
14	siRNA	PKN3	Liposomes	Pancreatic cancer	IV	NCT01808638	Completed
15	siRNA	HBV antigen	LNPs	Hepatitis B	IV	NCT02631096	Completed
16	siRNA	KSP and VEGF	LNPs	Solid tumors	IV	NCT01158079	Completed
17	siRNA	PCSK9	LNPs	Elevated LDL-Cholesterol	IV	NCT01437059	Completed
18	siRNA	Bcl-2	Gold NPs	GBM	IV	NCT03020017	Completed
19	siRNA	RRM2	Cyclodextrin polymer	Solid tumors	IV	NCT00689065	Terminated
20	siRNA	GO	LNPs	Primary hyperoxaluria type 1	IV	NCT02795325	Terminated
21	ASO	Grb-2	Liposomes	AML, ALL, MDS, CML	IV	NCT01159028	Active

Abbreviations: OX40L, ligand for OX40 receptor associated with tumor necrosis factor receptor superfamily; IT, intratumoral; IM, intramuscular; S-protein, spike protein from SARS-Cov-2; SARS-Cov-2, severe acute respiratory syndrome coronavirus 2; COVID-19, coronavirus disease-19; OTC, ornithine transcarbamylase; IV, intravenous; prM, pre-membrane protein; E, envelope protein; CMV; cytomegalovirus; MYC, family of proto-oncogenes; HSP47, gene encoding heat shock protein-47; PLK-1, polo-kinase-1; IA, intraarterial; CEBPA, gene encoding CCAAT/enhancer-binding protein alpha protein; TGF-β1, transforming growth factor beta 1; Cox-2, cyclooxygenase-2; KRAS, Kirsten rat sarcoma; SI, surgical implant; PKN3, protein kinase N3; KSP, kidney specific cadherin; VEGF, vascular endothelial growth factor; PCSK9; proprotein convertase subtilisin/kexin type 9; RRM2, ribonucleotide reductase M2; bcl-2, B-cell lymphoma 2; GO, glycolate oxidase; GBM, glioblastoma multiforme; Grb-2, growth factor receptor-bound protein 2; CML, chronic myeloid leukemia; AML, acute myeloid leukemia; ALL, acute lymphoblastic leukemia; MDS, myelodysplastic syndrome.

**Table 3:** A summary of advantages and disadvantages of lipid based and polymeric based vectors.

	Advantages	Disadvantages
**Cationic polymeric vectors**	• Control and sustained release kinetics	• Scale up and manufacturing is difficult
	• Functional group conjugation is achievable for active targeting [104]	• High cationic charge favors endosomal uptake but offers cellular toxicity
	• Better stability for the encapsulation of negatively charged nucleic acid cargo	• Poor clinical translation
	• Offers a wide range of polymeric systems based on temperature, pH, light sensitive, hydrolysis and enzyme degradation	
	• Optimization of chemical and physical properties is highly achievable by use of different polymer chemistries	
	• Offers biodegradable polymer options such as PLGA [97,145]	
	• Offers several natural polymers such as chitosan [97,141], hyaluronic acid and collagen [146]	
**Lipid based vectors**	• Easy scale up and manufacturing [28,29]	• Poor drug loading
	• Good pharmacokinetic and safety profile [147]	• Requires extensive formulation work to optimize ideal concentration of lipid components
	• Excellent clinical translation	
	• Allows conjugated ligands to be designed	

## References

[1] Kay M.A. (2011). State-of-the-art gene-based therapies: The road ahead. Nat. Rev. Genet..

[2] Quijano E., Bahal R., Ricciardi A., Saltzman W.M., Glazer P.M. (2017). Therapeutic peptide nucleic acids: Principles, limitations, and opportunities. Yale J. Biol. Med..

[3] Houa K.K., Panb H., Schlesingerc P.H., Wicklineb S.A. (2015). A Role for Peptides in Overcoming Endosomal Entrapment in siRNA Delivery—A Focus on Melittin. Biotechnol. Adv..

[4] Borgatti M., Romanelli M., Saviano M., Pedone C., Lampronti I., Breda L., Nastruzzi C., Bianchi N., Mischiati C., Gambari R. (2003). Resistance of decoy PNA-DNA chimeras to enzymatic degradation in cellular extracts and serum. Oncol. Res..

[5] Yin H., Kanasty R.L., Eltoukhy A.A., Vegas A.J., Dorkin J.R., Anderson D.G. (2014). Non-viral vectors for gene-based therapy. Nat. Rev. Genet..

[6] Shima M.S., Wang X., Ragan R., Kwon Y.J. (2010). Dynamics of Nucleic Acid/Cationic Polymer Complexation and Disassembly under Biologically Simulated Conditions Using In Situ Atomic Force Microscopy. Bone.

[7] Zhu L., Mahato R.I. (2010). Lipid and polymeric carrier-mediated nucleic acid delivery. Expert Opin. Drug Deliv..

[8] Longmire M.L., Choyke P.L., Kobayashi H. (2012). Clearance Properties of Nano-sized Particles and Molecules as Imagin Agents: Consideration and Caveats. Nanomedicine.

[9] Fang J., Nakamura H., Maeda H. (2011). The EPR effect: Unique features of tumor blood vessels for drug delivery, factors involved, and limitations and augmentation of the effect. Adv. Drug Deliv. Rev..

[10] Torchilin V.P. (2005). Recent advances with liposomes as pharmaceutical carriers. Nat. Rev. Drug Discov..

[11] Felgner P.L., Gadek T.R., Holm M., Roman R., Chan H.W., Wenz M., Northrop J.P., Ringold G.M., Danielsen M. (1987). Lipofection: A highly efficient, lipid-mediated DNA-transfection procedure. Proc. Natl. Acad. Sci. USA.

[12] Koltover I., Salditt T., Rädler J.O., Safinya C.R. (1998). An inverted hexagonal phase of cationic liposome-DNA complexes related to DNA release and delivery. Science.

[13] Gao H., Hui K.M. (2001). Synthesis of a novel series of cationic lipids that can act as efficient gene delivery vehicles through systematic heterocyclic substitution of cholesterol derivatives. Gene Ther..

[14] Lv H., Zhang S., Wang B., Cui S., Yan J. (2006). Toxicity of cationic lipids and cationic polymers in gene delivery. J. Control. Release.

[15] Hattori Y., Suzuki S., Kawakami S., Yamashita F., Hashida M. (2005). The role of dioleoylphosphatidylethanolamine (DOPE) in targeted gene delivery with mannosylated cationic liposomes via intravenous route. J. Control. Release.

[16] Dabkowska A.P., Barlow D.J., Hughes A.V., Campbell R.A., Quinn P.J., Lawrence M.J. (2012). The effect of neutral helper lipids on the structure of cationic lipid monolayers. J. R. Soc. Interface.

[17] Semple S.C., Chonn A., Cullis P.R. (1996). Influence of cholesterol on the association of plasma proteins with liposomes. Biochemistry.

[18] Thewalt J.L., Bloom M. (1992). Phosphatidylcholine: Cholesterol phase diagrams. Biophys. J..

[19] Barenholz Y. (2012). Doxil^®^—The first FDA-approved nano-drug: Lessons learned. J. Control. Release.

[20] Jokerst J., Lobovkina T., Zare R.N., Gambhir S.S. (2011). Nanoparticle PEGylation for imaging and therapy. Nanomedicine.

[21] Bao Y., Jin Y., Chivukula P., Zhang J., Liu Y., Liu J., Clamme J.P., Mahato R.I., Ng D., Ying W. (2013). Effect of PEGylation on biodistribution and gene silencing of siRNA/lipid nanoparticle complexes. Pharm. Res..

[22] Cheng X., Lee R.J. (2016). The role of helper lipids in lipid nanoparticles (LNPs) designed for oligonucleotide delivery. Adv. Drug Deliv. Rev..

[23] Filion M.C., Phillips N.C. (1997). Toxicity and immunomodulatory activity of liposomal vectors formulated with cationic lipids toward immune effector cells. Biochim. Biophys. Acta Biomembr..

[24] Knudsen K.B., Northeved H., Pramod Kumar E.K., Permin A., Gjetting T., Andresen T.L., Larsen S., Wegener K.M., Lykkesfeldt J., Jantzen K. (2015). In vivo toxicity of cationic micelles and liposomes. Nanomed. Nanotechnol. Biol. Med..

[25] Wheeler J.J., Palmer L., Ossanlou M., Maclachlan I., Graham R.W., Zhang Y.P., Hope M.J., Scherrer P., Cullis P.R. (1999). Stabilized plasmid-lipid particles: Construction and characterization. Gene Ther..

[26] Ambegia E., Ansell S., Cullis P., Heyes J., Palmer L., MacLachlan I. (2005). Stabilized plasmid-lipid particles containing PEG-diacylglycerols exhibit extended circulation lifetimes and tumor selective gene expression. Biochim. Biophys. Acta Biomembr..

[27] Cullis P.R. (2002). Stabilized plasmid-lipid particles for systemic gene therapy. Cell. Mol. Biol. Lett..

[28] Batzri S., Korn E.D. (1973). Single bilayer liposomes prepared without sonication. BBA Biomembr..

[29] Evers M.J.W., Kulkarni J.A., van der Meel R., Cullis P.R., Vader P., Schiffelers R.M. (2018). State-of-the-Art Design and Rapid-Mixing Production Techniques of Lipid Nanoparticles for Nucleic Acid Delivery. Small Methods.

[30] Jayaraman M., Ansell S.M., Mui B.L., Tam Y.K., Chen J., Du X., Butler D., Eltepu L., Matsuda S., Narayanannair J.K. (2012). Maximizing the potency of siRNA lipid nanoparticles for hepatic gene silencing in vivo. Angew. Chem. Int. Ed..

[31] Whitehead K.A., Dorkin J.R., Vegas A.J., Chang P.H., Veiseh O., Matthews J., Fenton O.S., Zhang Y., Olejnik K.T., Yesilyurt V. (2014). Degradable lipid nanoparticles with predictable in vivo siRNA delivery activity. Nat. Commun..

[32] Hafez I.M., Maurer N., Cullis P.R. (2001). On the mechanism whereby cationic lipids promote intracellular delivery of polynucleic acids. Gene Ther..

[33] Jirikowski G.F., Sanna P.P., Maciejewski-lenoir D., Bloom F.E., Jirikowski G.F., Sanna P.P., Maciejewski-lenoir D., Bloom F.E. (1992). Reversal of Diabetes Insipidus in Brattleboro Rats: Intrahypothalamic Injection of Vasopressin mRNA. Science.

[34] Pardia N., Tuyishimea S., Muramatsua H., Karikoa K., Muib B.L., Tamb Y.K., Maddenb T.D., Hopeb M.J., Weissmana D. (2015). Expression kinetics of nucleoside-modified mRNA delivered in lipid nanoparticles to mice by various routes. J. Control. Release.

[35] Kauffman K.J., Dorkin J.R., Yang J.H., Heartlein M.W., Derosa F., Mir F.F., Fenton O.S., Anderson D.G. (2015). Optimization of Lipid Nanoparticle Formulations for mRNA Delivery in Vivo with Fractional Factorial and Definitive Screening Designs. Nano Lett..

[36] Nabhan J.F., Wood K.M., Rao V.P., Morin J., Bhamidipaty S., Labranche T.P., Gooch R.L., Bozal F., Bulawa C.E., Guild B.C. (2016). Intrathecal delivery of frataxin mRNA encapsulated in lipid nanoparticles to dorsal root ganglia as a potential therapeutic for Friedreich’s ataxia. Sci. Rep..

[37] Sabnis S., Kumarasinghe E.S., Salerno T., Mihai C., Ketova T., Senn J.J., Lynn A., Bulychev A., McFadyen I., Chan J. (2018). A Novel Amino Lipid Series for mRNA Delivery: Improved Endosomal Escape and Sustained Pharmacology and Safety in Non-human Primates. Mol. Ther..

[38] Abbink P., Larocca R.A., De La Barrera R.A., Bricault C.A., Moseley E.T., Boyd M., Kirilova M., Li Z., Ng’ang’a D., Nanayakkara O. (2016). Protective Efficacy of Multiple Vaccine Platforms Against Zika Virus Challenge in Rhesus Monkeys. Science.

[39] Dowd K.A., Ko S.Y., Morabito K.M., Yang E.S., Pelc R.S., DeMaso C.R., Castilho L.R., Abbink P., Boyd M., Nityanandam R. (2016). Rapid development of a DNA vaccine for Zika virus. Science.

[40] Muthumani K., Griffin B.D., Agarwal S., Kudchodkar S.B., Reuschel E.L., Choi H., Kraynyak K.A., Duperret E.K., Keaton A.A., Chung C. (2016). In vivo protection against ZIKV infection and pathogenesis through passive antibody transfer and active immunisation with a prMEnv DNA vaccine. NPJ Vaccines.

[41] Ciaramella G., Shresta S., Pierson T.C., Salazar V., Dowd K.A., Richner J.M., Tang W.W., Himansu S., Butler S.L., Diamond M.S. (2017). Modified mRNA Vaccines Protect against Zika Virus Infection. Cell.

[42] Fire A., Xu S., Montgomery M.K., Kostas S.A., Driver S.E., Mello C.C. (1998). Potent and specific genetic interference by double-stranded RNA in *Caenorhabditis elegans*. Nature.

[43] Montgomery M.K., Xu S., Fire A. (1998). RNA as a target of double-stranded RNA-mediated genetic interference in Caenorhabditis elegans. Proc. Natl. Acad. Sci. USA.

[44] Elbashir S.M., Harborth J., Lendeckel W., Yalcin A., Weber K., Tuschl T. (2001). Duplexes of 21-nucleotide RNAs mediate RNA interference in cultured mammalian cells. Nature.

[45] Tomari Y., Zamore P.D. (2005). Perspective: Machines for RNAi. Genes Dev..

[46] Hammond S.M., Bernstein E., Beach D., Hannon G.J. (2000). An RNA-directed nuclease mediates post-transcriptional gene silencing in Drosophila cells. Nature.

[47] Bernstein E., Caudy A.A., Hammond S.M., Hannon G.J. (2001). Role for a bidentate ribonuclease in the initiation step of RNA interference. Nature.

[48] Ando Y., Coelho T., Berk J.L., Cruz M.W., Ericzon B.G., Ikeda S.I., Lewis W.D., Obici L., Planté-Bordeneuve V., Rapezzi C. (2013). Guideline of transthyretin-related hereditary amyloidosis for clinicians. Orphanet J. Rare Dis..

[49] Gertz M.A., Benson M.D., Dyck P.J., Grogan M., Coelho T., Cruz M., Berk J.L., Plante-Bordeneuve V., Schmidt H.H.J., Merlini G. (2015). Diagnosis, Prognosis, and Therapy of Transthyretin Amyloidosis. J. Am. Coll. Cardiol..

[50] Adams D., Gonzalez-Duarte A., O’Riordan W.D., Yang C.C., Ueda M., Kristen A.V., Tournev I., Schmidt H.H., Coelho T., Berk J.L. (2018). Patisiran, an RNAi therapeutic, for hereditary transthyretin amyloidosis. N. Engl. J. Med..

[51] Zhang X., Goel V., Robbie G.J. (2019). Pharmacokinetics of Patisiran, the First Approved RNA Interference Therapy in Patients With Hereditary Transthyretin-Mediated Amyloidosis. J. Clin. Pharmacol..

[52] Suhr O.B., Coelho T., Buades J., Pouget J., Conceicao I., Berk J., Schmidt H., Waddington-Cruz M., Campistol J.M., Bettencourt B.R. (2015). Efficacy and safety of patisiran for familial amyloidotic polyneuropathy: A phase II multi-dose study. Orphanet J. Rare Dis..

[53] Ball R.L., Hajj K.A., Vizelman J., Bajaj P., Whitehead K.A. (2018). Lipid Nanoparticle Formulations for Enhanced Co-delivery of siRNA and mRNA. Nano Lett..

[54] Joseph L., Robinson J.G. (2015). Proprotein Convertase Subtilisin/Kexin Type 9 (PCSK9) Inhibition and the Future of Lipid Lowering Therapy. Prog. Cardiovasc. Dis..

[55] Cohen J.C., Boerwinkle E., Mosley T.H., Hobbs H.H. (2006). Sequence Variations in PCSK9, Low LDL, and Protection against Coronary Heart Disease. N. Engl. J. Med..

[56] Suzuki Y., Hyodo K., Suzuki T., Tanaka Y., Kikuchi H., Ishihara H. (2017). Biodegradable lipid nanoparticles induce a prolonged RNA interference-mediated protein knockdown and show rapid hepatic clearance in mice and nonhuman primates. Int. J. Pharm..

[57] Zamecnik P.C., Stephenson M.L. (1978). Inhibition of Rous sarcoma virus replication and cell transformation by a specific oligodeoxynucleotide. Proc. Natl. Acad. Sci. USA.

[58] Juliano R.L. (2016). The delivery of therapeutic oligonucleotides. Nucleic Acids Res..

[59] Geary R.S., Norris D., Yu R., Bennett C.F. (2015). Pharmacokinetics, biodistribution and cell uptake of antisense oligonucleotides. Adv. Drug Deliv. Rev..

[60] Crooke S.T., Wang S., Vickers T.A., Shen W., Liang X.H. (2017). Cellular uptake and trafficking of antisense oligonucleotides. Nat. Biotechnol..

[61] Glazier D.A., Liao J., Roberts B., Li X., Yang K., Stevens C.M., Tang W. (2020). Chemical Synthesis and Biological Application of Modified Oligonucleotides. Bioconjug. Chem..

[62] Mathew V., Wang A.K. (2019). Inotersen: New promise for the treatment of hereditary transthyretin amyloidosis. Drug Des. Devel. Ther..

[63] Hagedorn P.H., Persson R., Funder E.D., Albæk N., Diemer S.L., Hansen D.J., Møller M.R., Papargyri N., Christiansen H., Hansen B.R. (2018). Locked nucleic acid: Modality, diversity, and drug discovery. Drug Discov. Today.

[64] Summerton J., Weller D. (1997). Morpholino antisense oligomers: Design, preparation, and properties. Antisense Nucleic Acid Drug Dev..

[65] Nielsen P.E. (1994). Peptide nucleic acid (PNA). A structural DNA mimic. Mater. Res. Soc. Symp. Proc..

[66] Shen X., Corey D.R. (2018). Chemistry, mechanism and clinical status of antisense oligonucleotides and duplex RNAs. Nucleic Acids Res..

[67] Dominski Z., Kole R. (1993). Restoration of correct splicing in thalassemic pre-mRNA by antisense oligonucleotides. Proc. Natl. Acad. Sci. USA.

[68] Havens M.A., Hastings M.L. (2016). Splice-switching antisense oligonucleotides as therapeutic drugs. Nucleic Acids Res..

[69] Knudsen H., Nielsen P.E. (1996). Antisense properties of duplex- and triplex-forming PNAs. Nucleic Acids Res..

[70] Dean D.A. (2000). Peptide nucleic acids: Versatile tools for gene therapy strategies. Adv. Drug Deliv. Rev..

[71] Summerton J. (1999). Morpholino antisense oligomers: The case for an RNase H-independent structural type. Biochim. Biophys. Acta.

[72] Yang L., Ma F., Liu F., Chen J., Zhao X., Xu Q. (2020). Efficient Delivery of Antisense Oligonucleotides Using Bioreducible Lipid Nanoparticles In Vitro and In Vivo. Mol. Ther. Nucleic Acids.

[73] Gleave M.E., Monia B.P. (2005). Antisense therapy for cancer. Nat. Rev. Cancer.

[74] Cheng X., Liu Q., Li H., Kang C., Liu Y., Guo T., Shang K., Yan C., Cheng G., Lee R.J. (2017). Lipid Nanoparticles Loaded with an Antisense Oligonucleotide Gapmer Against Bcl-2 for Treatment of Lung Cancer. Pharm. Res..

[75] Boussif O., Lezoualc’h F., Zanta M.A., Mergny M.D., Scherman D., Demeneixt B., Behr J.–P. (1995). A Versatile Vector for Gene and Oligonucleotide Transfer into Cells in Culture and in vivo: Polyethylenimine. Proc. Natl. Acad. Sci. USA.

[76] Wu G.Y., Wu C.H. (1987). Receptor-mediated in vitro gene transformation by a soluble DNA carrier system. J. Biol. Chem..

[77] Behr J.P. (1997). The proton sponge: A trick to enter cells the viruses did not exploit. Chimia.

[78] Vermeulen L.M.P., Brans T., Samal S.K., Dubruel P., Demeester J., De Smedt S.C., Remaut K., Braeckmans K. (2018). Endosomal Size and Membrane Leakiness Influence Proton Sponge-Based Rupture of Endosomal Vesicles. ACS Nano.

[79] Wojnilowicz M., Glab A., Bertucci A., Caruso F., Cavalieri F. (2019). Super-resolution Imaging of Proton Sponge-Triggered Rupture of Endosomes and Cytosolic Release of Small Interfering RNA. ACS Nano.

[80] Godbey W.T., Wu K.K., Mikos A.G. (1999). Size matters: Molecular weight affects the efficiency of poly(ethylenimine) as a gene delivery vehicle. J. Biomed. Mater. Res..

[81] Fischer D., Bieber T., Li Y., Elsässer H.P., Kissel T. (1999). A novel non-viral vector for DNA delivery based on low molecular weight, branched polyethylenimine: Effect of molecular weight on transfection efficiency and cytotoxicity. Pharm. Res..

[82] Gao X., Yao L., Song Q., Zhu L., Xia Z., Xia H., Jiang X., Chen J., Chen H. (2011). The association of autophagy with polyethylenimine-induced cytotoxity in nephritic and hepatic cell lines. Biomaterials.

[83] Green J.J., Langer R., Anderson D.G. (2008). A combinatorial polymer library approach yields insight into nonviral gene delivery. Acc. Chem. Res..

[84] Zhou J., Liu J., Cheng C.J., Patel T.R., Weller C.E., Piepmeier J.M., Jiang Z., Saltzman W.M. (2012). Biodegradable poly(amine-co-ester) terpolymers for targeted gene delivery. Nat. Mater..

[85] Kauffman A.C., Piotrowski-Daspit A.S., Nakazawa K.H., Jiang Y., Datye A., Saltzman W.M. (2018). Tunability of Biodegradable Poly(amine-co-ester) Polymers for Customized Nucleic Acid Delivery and Other Biomedical Applications. Biomacromolecules.

[86] Obata Y., Saito S., Takeda N., Takeoka S. (2009). Plasmid DNA-encapsulating liposomes: Effect of a spacer between the cationic head group and hydrophobic moieties of the lipids on gene expression efficiency. Biochim. Biophys. Acta Biomembr..

[87] Piest M., Engbersen J.F.J. (2010). Effects of charge density and hydrophobicity of poly(amido amine)s for non-viral gene delivery. J. Control. Release.

[88] Patel A.K., Kaczmarek J.C., Bose S., Kauffman K.J., Mir F., Heartlein M.W., DeRosa F., Langer R., Anderson D.G. (2019). Inhaled Nanoformulated mRNA Polyplexes for Protein Production in Lung Epithelium. Adv. Mater..

[89] Jiang Y., Gaudin A., Zhang J., Agarwal T., Song E., Kauffman A.C., Tietjen G.T., Wang Y., Jiang Z., Cheng C.J. (2018). A “top-down” approach to actuate poly(amine-co-ester) terpolymers for potent and safe mRNA delivery. Biomaterials.

[90] Jiang Y., Lu Q., Wang Y., Xu E., Ho A., Singh P., Wang Y., Jiang Z., Yang F., Tietjen G.T. (2020). Quantitating Endosomal Escape of a Library of Polymers for mRNA Delivery. Nano Lett..

[91] Cui J., Qin L., Zhang J., Abrahimi P., Li H., Li G., Tietjen G.T., Tellides G., Pober J.S., Mark Saltzman W. (2017). Ex vivo pretreatment of human vessels with siRNA nanoparticles provides protein silencing in endothelial cells. Nat. Commun..

[92] Cui J., Piotrowski-Daspit A.S., Zhang J., Shao M., Bracaglia L.G., Utsumi T., Seo Y.E., DiRito J., Song E., Wu C. (2019). Poly(amine-co-ester) nanoparticles for effective Nogo-B knockdown in the liver. J. Control. Release.

[93] Munier S., Messai I., Delair T., Verrier B., Ataman-Önal Y. (2005). Cationic PLA nanoparticles for DNA delivery: Comparison of three surface polycations for DNA binding, protection and transfection properties. Colloids Surf. B Biointerfaces.

[94] Morin G.B. (1989). The human telomere terminal transferase enzyme is a ribonucleoprotein that synthesizes TTAGGG repeats. Cell.

[95] Holt S.E., Shay J.W., Wright W.E. (1996). Refining the telomere-telomerase hypothesis of aging and cancer. Nat. Biotechnol..

[96] Pitts A.E., Corey D.R. (1998). Inhibition of human telomerase by 2′-O-methyl-RNA. Proc. Natl. Acad. Sci. USA.

[97] Nafee N., Taetz S., Schneider M., Schaefer U.F., Lehr C.M. (2007). Chitosan-coated PLGA nanoparticles for DNA/RNA delivery: Effect of the formulation parameters on complexation and transfection of antisense oligonucleotides. Nanomed. Nanotechnol. Biol. Med..

[98] Feinbaum R., Ambros V., Lee R. (1993). The C. elegans Heterochronic Gene lin-4 Encodes Small RNAs with Antisense Complementarity to lin-14. Cell.

[99] Friedman R.C., Farh K.K.H., Burge C.B., Bartel D.P. (2009). Most mammalian mRNAs are conserved targets of microRNAs. Genome Res..

[100] Catalanotto C., Cogoni C., Zardo G. (2016). MicroRNA in control of gene expression: An overview of nuclear functions. Int. J. Mol. Sci..

[101] Baumann V., Winkler J. (2014). MiRNA-based therapies: Strategies and delivery platforms for oligonucleotide and non-oligonucleotide agents. Future Med. Chem..

[102] Peng Y., Croce C.M. (2016). The role of microRNAs in human cancer. Signal Transduct. Target. Ther..

[103] Seo Y.E., Suh H.W., Bahal R., Josowitz A., Zhang J., Song E., Cui J., Noorbakhsh S., Jackson C., Bu T. (2019). Nanoparticle-mediated intratumoral inhibition of miR-21 for improved survival in glioblastoma. Biomaterials.

[104] Shmueli R.B., Anderson D.G., Green J.J. (2010). Electrostatic surface modifications to improve gene delivery. Expert Opin. Drug Deliv..

[105] Lee J.S., Green J.J., Love K.T., Sunshine J., Langer R., Anderson D.G. (2009). Gold, poly(β-amino ester) nanoparticles for small interfering RNA delivery. Nano Lett..

[106] Runcie K., Budman D.R., John V., Seetharamu N. (2018). Bi-specific and tri-specific antibodies- the next big thing in solid tumor therapeutics. Mol. Med..

[107] Tsuchikama K., An Z. (2018). Antibody-drug conjugates: Recent advances in conjugation and linker chemistries. Protein Cell.

[108] Kaplon H., Muralidharan M., Schneider Z., Reichert J.M. (2020). Antibodies to watch in 2020. MAbs.

[109] Johnston M.C., Scott C.J. (2018). Antibody conjugated nanoparticles as a novel form of antibody drug conjugate chemotherapy. Drug Discov. Today Technol..

[110] Okamoto A., Asai T., Hirai Y., Shimizu K., Koide H., Minamino T., Oku N. (2018). Systemic Administration of siRNA with Anti-HB-EGF Antibody-Modified Lipid Nanoparticles for the Treatment of Triple-Negative Breast Cancer. Mol. Pharm..

[111] Soutschek J., Akinc A., Bramlage B., Charisse K., Constien R., Donoghue M., Elbashir S., Gelck A., Hadwiger P., Harborth J. (2004). Therapeutic silencing of an endogenous gene by systemic administration of modified siRNAs. Nature.

[112] Ambardekar V.V., Han H., Varney M.L., Vinogradov S.V., Singh R.K., Vetro J.A. (2011). The modification of siRNA with 3′ cholesterol to increase nuclease protection and suppression of native mRNA by select siRNA polyplexes. Biomaterials.

[113] Alnylam Alnylam^®^ Development Pipeline of Investigational RNAi Therapeutics. https://www.alnylam.com/alnylam-rnai-pipeline/2020.

[114] Wooddell C.I., Rozema D.B., Hossbach M., John M., Hamilton H.L., Chu Q., Hegge J.O., Klein J.J., Wakefield D.H., Oropeza C.E. (2013). Hepatocyte-targeted RNAi therapeutics for the treatment of chronic hepatitis B virus infection. Mol. Ther..

[115] Wooddell C.I., Yuen M.F., Chan H.L.Y., Gish R.G., Locarnini S.A., Chavez D., Ferrari C., Given B.D., Hamilton J., Kanner S.B. (2017). Rnai-based treatment of chronically infected patients and chimpanzees reveals that integrated hepatitis b virus DNA is a source of hbsag. Sci. Transl. Med..

[116] Wu J., Nantz M.H., Zern M.A. (2002). Targeting hepatocytes for drug and gene delivery: Emerging novel approaches and applications. Front. Biosci..

[117] de Paula Brandão P.R., Titze-de-Almeida S.S., Titze-de-Almeida R. (2020). Leading RNA Interference Therapeutics Part 2: Silencing Delta-Aminolevulinic Acid Synthase 1, with a Focus on Givosiran. Mol. Diagnosis Ther..

[118] Hill A., Taubel J., Bush J., Borodovsky A., Kawahata N., Mclean H., Powell C., Chaturvedi P., Warner G., Garg P. (2015). A Subcutaneously Administered Investigational RNAi Therapeutic (ALN-CC5) Targeting Complement C5 for Treatment of PNH and Complement-Mediated Diseases: Interim Phase 1 Study Results. Blood.

[119] Hill A., Valls A.G., Griffin M., Munir T., Borodovsky A., Kawahata N., Mclean H., Shi K., Partisano A.M., Kim J. (2016). A subcutaneously administered investigational RNAi therapeutic (ALN-CC5) targeting complement C5 for treatment of PNH and complement-mediated diseases: Preliminary phase 1/2 study results in patients with PNH. Blood.

[120] Liebow A., Li X., Racie T., Hettinger J., Bettencourt B.R., Najafian N., Haslett P., Fitzgerald K., Holmes R.P., Erbe D. (2017). An investigational RNAi therapeutic targeting glycolate oxidase reduces oxalate production in models of primary hyperoxaluria. J. Am. Soc. Nephrol..

[121] Judge D.P., Kristen A.V., Grogan M., Maurer M.S., Falk R.H., Hanna M., Gillmore J., Garg P., Vaishnaw A.K., Harrop J. (2020). Phase 3 Multicenter Study of Revusiran in Patients with Hereditary Transthyretin-Mediated (hATTR) Amyloidosis with Cardiomyopathy (ENDEAVOUR). Cardiovasc. Drugs Ther..

[122] Pasi K.J., Georgiev P., Mant T., Lissitchkov T., Creagh M.D., Bevan D., Austin S., Hay C.R., Hegemann I., Kazmi R. (2016). Fitusiran, an Investigational RNAi Therapeutic Targeting Antithrombin for the Treatment of Hemophilia: Updated Results from a Phase 1 and Phase 1/2 Extension Study in Patients with Inhibitors. Blood.

[123] Pipe S., Ragni M.V., Négrier C., Yu Q., Bajwa N., Caminis J., Mei B., Andersson S.R. (2019). Fitusiran, an RNAi Therapeutic Targeting Antithrombin to Restore Hemostatic Balance in Patients with Hemophilia a or B with or without Inhibitors: Management of Acute Bleeding Events. Blood.

[124] Fitzgerald K., White S., Borodovsky A., Bettencourt B.R., Strahs A., Clausen V., Wijngaard P., Horton J.D., Taubel J., Brooks A. (2017). A highly durable RNAi therapeutic inhibitor of PCSK9. N. Engl. J. Med..

[125] Ray K.K., Landmesser U., Leiter L.A., Kallend D., Dufour R., Karakas M., Hall T., Troquay R.P.T., Turner T., Visseren F.L.J. (2017). Inclisiran in patients at high cardiovascular risk with elevated LDL cholesterol. N. Engl. J. Med..

[126] Ray K.K., Wright R.S., Kallend D., Koenig W., Leiter L.A., Raal F.J., Bisch J.A., Richardson T., Jaros M., Wijngaard P.L.J. (2020). Two Phase 3 Trials of Inclisiran in Patients with Elevated LDL Cholesterol. N. Engl. J. Med..

[127] Graham M.J., Lee R.G., Brandt T.A., Tai L.J., Fu W., Peralta R., Yu R., Hurh E., Paz E., McEvoy B.W. (2017). Cardiovascular and metabolic effects of ANGPTL3 antisense oligonucleotides. N. Engl. J. Med..

[128] Han K., Cremer J., Elston R., Oliver S., Baptiste-Brown S., Chen S., Gardiner D., Davies M., Saunders J., Hamatake R. (2019). A Randomized, Double-Blind, Placebo-Controlled, First-Time-in-Human Study to Assess the Safety, Tolerability, and Pharmacokinetics of Single and Multiple Ascending Doses of GSK3389404 in Healthy Subjects. Clin. Pharmacol. Drug Dev..

[129] McCaleb M., Hughes S., Greary R., Monia B., Grossman T. (2018). Pharmacodynamic efficacy of IONIS-FB-LRX, a complement factor B antisense oligonucleotide, in a phase 1 clinical study. Mol. Immunol..

[130] Ferrone J.D., Bhattacharjee G., Revenko A.S., Zanardi T.A., Warren M.S., Derosier F.J., Viney N.J., Pham N.C., Kaeser G.E., Baker B.F. (2019). IONIS-PKK Rx a Novel Antisense Inhibitor of Prekallikrein and Bradykinin Production. Nucleic Acid Ther..

[131] Rozema D.B., Lewis D.L., Wakefield D.H., Wong S.C., Klein J.J., Roesch P.L., Bertin S.L., Reppen T.W., Chu Q., Blokhin A.V. (2007). Dynamic PolyConjugates for targeted in vivo delivery of siRNA to hepatocytes. Proc. Natl. Acad. Sci. USA.

[132] Wong S.C., Klein J.J., Hamilton H.L., Chu Q., Frey C.L., Trubetskoy V.S., Hegge J., Wakefield D., Rozema D.B., Lewis D.L. (2012). Co-injection of a targeted, reversibly masked endosomolytic polymer dramatically improves the efficacy of cholesterol-conjugated small interfering RNAs in vivo. Nucleic Acid Ther..

[133] Li H., Yang Y., Hong W., Huang M., Wu M., Zhao X. (2020). Applications of genome editing technology in the targeted therapy of human diseases: Mechanisms, advances and prospects. Signal Transduct. Target. Ther..

[134] Gaj T., Gersbach C.A., Barbas C.F. (2013). ZFN, TALEN, and CRISPR/Cas-based methods for genome engineering. Trends Biotechnol..

[135] O’Driscoll M., Jeggo P.A. (2006). The role of double-strand break repair—Insights from human genetics. Nat. Rev. Genet..

[136] Carroll D. (2011). Genome engineering with zinc-finger nucleases. Genetics.

[137] Joung J.K., Sander J.D. (2013). TALENs: A widely applicable technology for targeted genome editing. Nat. Rev. Mol. Cell Biol..

[138] Mali P., Yang L., Esvelt K.M., Aach J., Guell M., DiCarlo J.E., Norville J.E., Church G.M. (2013). RNA-guided human genome engineering via Cas9. Science.

[139] Cong L., Ran F.A., Cox D., Lin S., Barretto R., Hsu P.D., Wu X., Jiang W., Marraffini L.A. (2013). Multiplex Genome Engineering Using CRISPR/Cas Systems. Science.

[140] Mahiny A.J., Dewerth A., Mays L.E., Alkhaled M., Mothes B., Malaeksefat E., Loretz B., Rottenberger J., Brosch D.M., Reautschnig P. (2015). In vivo genome editing using nuclease-encoding mRNA corrects SP-B deficiency. Nat. Biotechnol..

[141] Conway A., Mendel M., Kim K., McGovern K., Boyko A., Zhang L., Miller J.C., DeKelver R.C., Paschon D.E., Mui B.L. (2019). Non-viral Delivery of Zinc Finger Nuclease mRNA Enables Highly Efficient In Vivo Genome Editing of Multiple Therapeutic Gene Targets. Mol. Ther..

[142] Sun W., Ji W., Hall J.M., Hu Q., Wang C., Beisel C.L., Gu Z. (2015). Efficient Delivery of CRISPR-Cas9 for Genome Editing via Self-Assembled DNA Nanoclews. Angew. Chem. Int. Ed. Engl..

[143] Jiang C., Mei M., Li B., Zhu X., Zu W., Tian Y., Wang Q., Guo Y., Dong Y., Tan X. (2017). A non-viral CRISPR/Cas9 delivery system for therapeutically targeting HBV DNA and pcsk9 in vivo. Cell Res..

[144] Jia N., Ye Y., Wang Q., Zhao X., Hu H., Chen D., Qiao M. (2017). Preparation and evaluation of poly(L-histidine) based pH-sensitive micelles for intracellular delivery of doxorubicin against MCF-7/ADR cells. Asian J. Pharm. Sci..

[145] Rezvantalab S., Drude N.I., Moraveji M.K., Güvener N., Koons E.K., Shi Y., Lammers T., Kiessling F. (2018). PLGA-based nanoparticles in cancer treatment. Front. Pharmacol..

[146] Tong X., Pan W., Su T., Zhang M., Dong W., Qi X. (2020). Recent advances in natural polymer-based drug delivery systems. React. Funct. Polym..

[147] Doktorovová S., Kovačević A.B., Garcia M.L., Souto E.B. (2016). Preclinical safety of solid lipid nanoparticles and nanostructured lipid carriers: Current evidence from in vitro and in vivo evaluation. Eur. J. Pharm. Biopharm..

